# Cancer outcomes and biological mechanisms among patients with type 2 diabetes mellitus using glucagon-like peptide-1 receptor agonists: a systematic review and meta-analysis

**DOI:** 10.3389/fonc.2026.1859759

**Published:** 2026-07-14

**Authors:** Ejike Daniel Eze, Leopold Ntakirutimana, Swase Dominic Terkimbi, Muluken Walle, Abdullahi Hussein Umar, Diresibachew Haile Wondimu, Makinde Vincent Olubiyi, Onyinye Cynthia Okeke, Olufunke Onaadepo, Jimoh Abdulazeez, David Chibuike Ikwuka, Elemi John Ani, Abdullateef Isiaka Alagbonsi

**Affiliations:** 1 Department of Physiology, School of Medicine and Pharmacy, College of Medicine and Health Sciences, University of Rwanda, Huye, Southern Province, Rwanda; 2 Department of Pharmacy, Faculty of Health Science, Victoria University, Kampala, Uganda

**Keywords:** cancer, glucagon-like peptide-1 receptor agonists, meta-analysis, prevalence, type 2 diabetes mellitus

## Abstract

**Introduction:**

While glucagon-like peptide-1 receptor agonists (GLP-1RAs) provide metabolic and cardiovascular benefits in type 2 diabetes mellitus (T2DM) patients, uncertainty remains regarding their association with cancer outcomes and the biological mechanisms by which they influence tumor development. This systematic review and meta-analysis evaluated overall and site-specific cancer outcomes among adults with T2DM using GLP-1RAs.

**Methodology:**

The review was conducted in accordance with PRISMA 2020 guidelines. PubMed, Scopus, and Web of Science were searched on 8 June 2026. Eligible studies included adults with T2DM exposed to GLP-1RAs and reported cancer-related outcomes. Hazard ratios (HRs) were pooled using the Generic Inverse Variance method under a random-effects model.

**Results:**

The pooled analysis showed a significant reduction in overall cancer risk among GLP-1RAs users (HR = 0.86, 95% CI: 0.74–0.99; p = 0.04). Cancer-specific analyses showed significant reductions in pancreatic, colorectal, endometrial, ovarian, hepatocellular, esophageal, and gastric cancers, while no significant associations were observed for thyroid, breast, kidney, or prostate cancers. Mechanistic evidence was limited and mostly indirect, with few studies assessing molecular pathways related to inflammation, cellular proliferation, apoptosis, and metabolic regulation.

**Discussion:**

Overall, GLP-1RAs were not associated with an increased cancer risk and may be linked to a reduced risk for certain cancer types. However, substantial heterogeneity, limited mechanistic validation, and reliance on observational evidence warrant cautious interpretation.

## 1 Introduction

Diabetes mellitus (DM), a metabolic disorder characterized by impaired insulin secretion and/or action leading to chronic hyperglycemia, causes disturbances in carbohydrate, lipid, and protein metabolisms (1). In 2024, approximately 588.7 million adults aged 20–79 years (about 1 in 9 globally) were living with DM, with over 40% unaware of their condition (2). The global burden is projected to rise by 46%, reaching approximately 853 million adults by 2050. More than 90% of these cases are type 2 diabetes mellitus (T2DM), driven by urbanization, ageing populations, sedentary lifestyles, overweight, and genetic susceptibility (2, 3). In Africa, an estimated 24.6 million people were living with DM in 2024, with projections reaching 59.5 million by 2050. In Rwanda, the prevalence of DM is estimated at 1.85%, which is lower than in Tanzania (9.2%), the Democratic Republic of Congo (6.3%), Ethiopia (3.6%), Burundi (3.6%), and Kenya (2.8%), but slightly higher than in Uganda (1.7%) (3). The rapid global rise in T2DM has led to expanded use of newer glucose-lowering therapies, particularly glucagon-like peptide-1 receptor agonists (GLP-1RAs), which are prescribed for glycemic control and cardiometabolic risk reduction.

The GLP-1RAs, including semaglutide, liraglutide, dulaglutide, and exenatide, offer benefits beyond glucose lowering (4), as they enhance glucose-dependent insulin secretion, suppress glucagon release, delay gastric emptying, and promote satiety. Their clinical effects include improved glycemic control, weight reduction, and decreased major adverse cardiovascular events in high-risk patients (5). Despite these therapeutic advantages, concerns regarding a potential association between GLP-1RAs and cancer have generated scientific and regulatory debates (6). Early preclinical studies in rodents demonstrated C-cell hyperplasia and medullary thyroid carcinoma, leading to safety warnings and post-marketing surveillance requirements (7). However, human data remain inconsistent. Large population-based cohort studies and regulatory evaluations have generally not demonstrated a significant increase in overall cancer incidence among GLP-1RAs users. In contrast, some smaller studies and subgroup analyses have reported possible elevated risks of thyroid, pancreatic, or gastrointestinal cancers (8).

Mechanistically, GLP-1 receptor activation influences intracellular signaling pathways involved in cell proliferation, survival, and apoptosis (9). The GLP-1RAs modulate cyclic adenosine monophosphate (cAMP)–protein kinase A (PKA), phosphoinositide 3-kinase (PI3K)/protein kinase B (Akt)/mTOR pathways, and nuclear factor-κB (NF-κB)–mediated inflammatory cascades, all of which are implicated in tumor biology. In pancreatic tissue, GLP-1RAs have been associated with β-cell proliferation and ductal remodeling (10). Although human thyroid C-cells express minimal GLP-1 receptors, theoretical concerns include altered calcitonin secretion and proliferative stimulation under specific genetic or hormonal contexts. Furthermore, GLP-1RAs interact with insulin and IGF-1 signaling pathways known to influence oncogenesis (11).

T2DM is ordinarily associated with an increased risk of several cancers, including pancreatic, liver, endometrial, colorectal, and breast cancers (12). These associations are influenced by factors such as obesity, chronic inflammation, hyperinsulinemia, viral infections (HBV, HCV, and HIV), and environmental exposures (1). Consequently, distinguishing the independent effect of GLP-1RAs from the underlying cancer risk associated with T2DM remains challenging. Therefore, this systematic review and meta-analysis aimed to synthesize the evidence on cancer outcomes among patients with T2DM using GLP-1RAs and to evaluate the available evidence on the potential mechanisms through which GLP-1RAs may influence tumor biology. By integrating epidemiological and mechanistic evidence, this review aims to clarify the current state of knowledge and identify areas requiring further investigation.

## 2 Methods

### 2.1 Study design

This study is a systematic review and meta-analysis conducted and reported in accordance with the Preferred Reporting Items for Systematic Reviews and Meta-Analyses (PRISMA) 2020 guidelines (13). The PRISMA 2020 checklist is provided as Supplementary File S1.

### 2.2 Search strategy

A comprehensive and structured search was conducted on 8^th^ June, 2026 for studies spanning 2015 up to 31^st^ May, 2026, using three major electronic databases (PubMed, Scopus, and Web of Science). The search period corresponds to the modern era of widespread clinical use of GLP-1RAs and the availability of large-scale safety and outcome data. Search strategies combined controlled vocabulary terms and free-text keywords relating to GLP-1RAs, cancer, and T2DM. The approach was adapted for each database to achieve optimal sensitivity. For PubMed, the core search string combined terms for the exposure (for example, “GLP-1 receptor agonist”, GLP-1RAs”, liraglutide, semaglutide, dulaglutide, exenatide, lixisenatide, tirzepatide), outcome (cancer, malignancy, neoplasm, carcinoma, tumor), and the underlying condition (type 2 diabetes or T2DM). For Web of Science and Scopus, the search used a similar conceptual structure but adapted to the database syntax, combining GLP-1RAs–related terms (GLP-1RAs, liraglutide, semaglutide, dulaglutide, exenatide, lixisenatide, tirzepatide) with cancer-related terms (cancer, malignan, neoplasm, tumor) and DM-related terms. The search strings used for each database and the number of retrieved records are presented in **Supplementary File S2**.

### 2.3 Eligibility criteria

The eligibility criteria were developed using the PICO (Population, Intervention, Comparison, Outcome) framework (**Table 1**). Studies were included if they involved adults (≥18 years) with T2DM, evaluated exposure to GLP-1RAs, included a comparator group comprising non-users of GLP-1RAs or users of other glucose-lowering therapies, and reported cancer-related outcomes, including overall or site-specific cancer risk. Eligible study designs included randomized controlled trials, cohort studies, and case–control studies. Studies were excluded if they were animal or *in vitro* studies, reviews, systematic reviews, meta-analyses, editorials, commentaries, conference abstracts, case reports, or case series. Studies that did not involve adults with T2DM, did not evaluate GLP-1RAs exposure, or did not report cancer-related outcomes were also excluded.

**Table 1:** PICO elements guiding the research objectives of the review.

PICO element	Inclusion criteria
Population (P)	Adults (≥18 years) with T2DM
Intervention (I)	Treatment with GLP-1RAs
Comparison (C)	Non-use of GLP-1RAs or use of other glucose-lowering therapies
Outcome (O)	Cancer outcomes, including incidence, progression, mortality, and tumor-specific outcomes; and/or reported biological or mechanistic evidence related to cancer processes

GLP-1RAs, glucagon-like peptide-1 receptor agonists; T2DM, type-2 diabetes mellitus.

### 2.4 Study selection

All retrieved records were exported as CSV files and imported into Rayyan for screening and duplicate management. Duplicate records identified by the software were verified manually and removed. Two reviewers independently screened titles and abstracts against the predefined eligibility criteria. Full texts of potentially eligible studies were then retrieved and assessed for inclusion. Studies meeting all eligibility criteria were included in the systematic review. Studies that provided sufficient quantitative data for effect estimation were subsequently included in the meta-analysis. Disagreements at any stage of the selection process were resolved through discussion and, where necessary, consultation with a third reviewer.

### 2.5 Data extraction

Data were extracted independently by two reviewers using a standardized data extraction form developed *a priori*. Extracted information included study characteristics (first author, year of publication, country, study design, sample size, and follow-up duration), participant characteristics (mean or median age and baseline clinical characteristics where reported), and exposure characteristics (type of GLP-1RAs and comparator therapy). For each study, cancer-related outcomes were extracted, including overall cancer incidence and site-specific cancer outcomes. Any disagreements between reviewers were resolved through discussion and verification against the original publications. The complete data extraction form is provided in Supplementary File S3.

### 2.6 Risk of bias assessment

Risk of bias was assessed using the Risk of Bias in Non-randomized Studies of Interventions (ROBINS-I) tool. This instrument evaluates bias across key domains, including confounding, participant selection, classification of interventions, deviations from intended interventions, missing data, measurement of outcomes, and selection of reported results. Each domain was rated as low, moderate, serious, critical, or no information, and an overall risk-of-bias judgment was assigned to each included study.

### 2.7 Data synthesis and meta-analysis

Meta-analyses were performed using Review Manager (RevMan) version 5.4. Hazard ratios (HRs) and corresponding 95% confidence intervals (CIs) were pooled using the Generic Inverse Variance method. Random-effects models were applied for all quantitative analyses. Cancer-specific analyses were conducted according to cancer type, and fewer than three studies (gallbladder cancer, bladder cancer, multiple myeloma, and meningioma) were not quantitatively synthesized due to insufficient data for a reliable meta-analysis. Findings for these outcomes were therefore summarized narratively and summarized in a table.

## 3 Results

### 3.1 Search results

**Figure 1:**
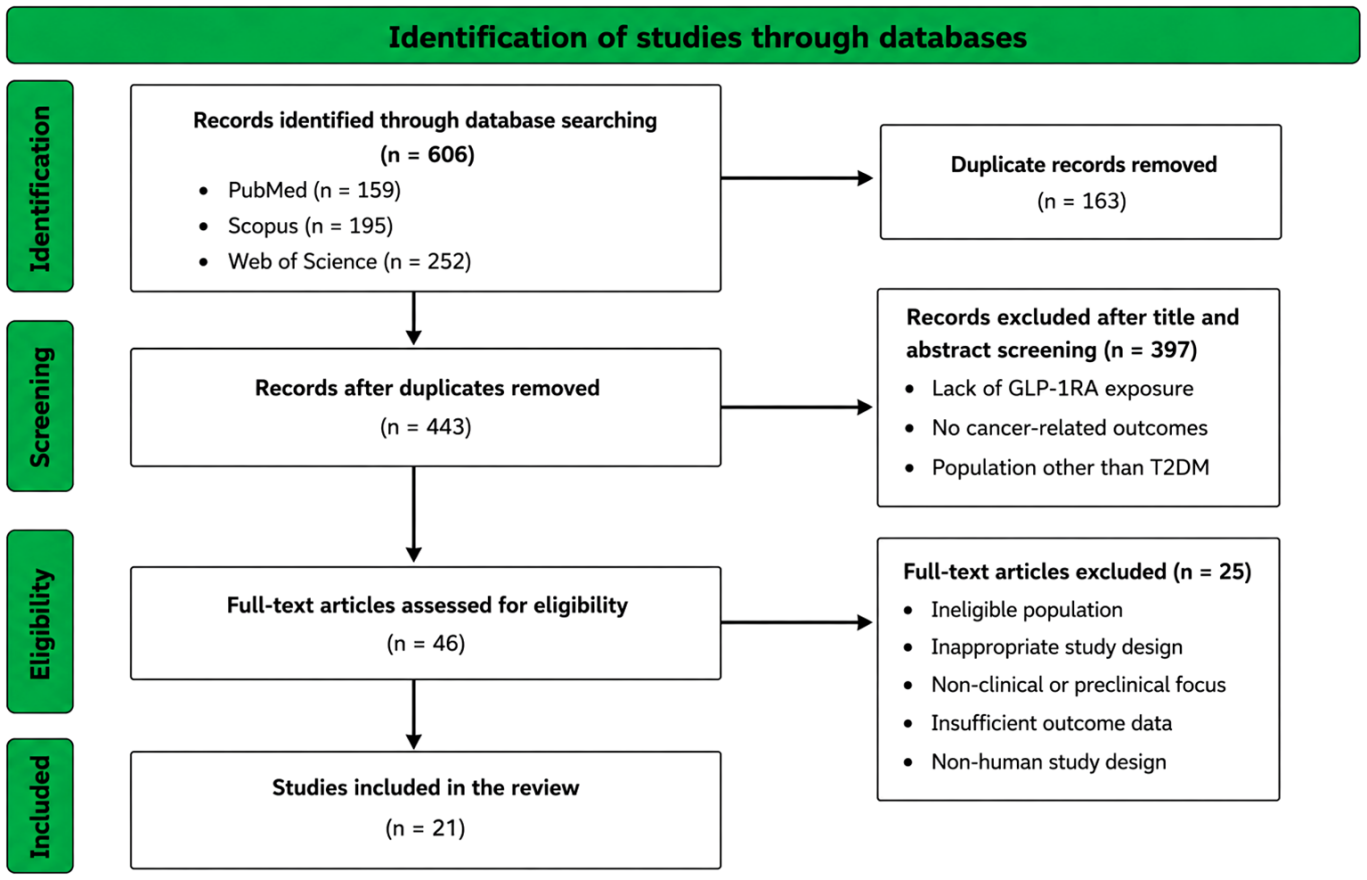
PRISMA flow chart of the article screening and selection process.

The database search identified 606 records, comprising PubMed (n = 159), Scopus (n = 195), and Web of Science (n = 252). After removal of 163 duplicates, 443 unique records remained for title and abstract screening. Of these, 397 articles were excluded because they did not meet the eligibility criteria, including lack of GLP-1RAs exposure, absence of cancer-related outcomes, or inclusion of populations other than patients with T2DM. The remaining 46 articles underwent full-text assessment. Following a detailed review, 25 studies were excluded due to ineligible populations, inappropriate study designs, non-clinical or preclinical focus, insufficient outcome data, or non-human study designs. Ultimately, 21 studies met the inclusion criteria and were included in the systematic review and meta-analysis (**Figure 1**).

### 3.2 Study characteristics

A total of 21 studies were included in this systematic review and meta-analysis (**Table 2**). Most studies originated from the United States (n = 13), followed by Israel (n = 2), while France, South Korea, and the United Kingdom each contributed one study. In addition, three studies were conducted using multinational or global datasets. Regarding publication year, the largest number of studies were published in 2025 (n = 8), followed by 2024 (n = 7), 2026 (n = 3), 2023 (n = 1), and 2016 (n = 1). Most studies employed retrospective cohort designs, with several incorporating propensity score matching (PSM), target-trial emulation, or active-comparator new-user approaches. The included studies enrolled large populations of adults with T2DM and evaluated cancer outcomes associated with GLP-1RAs. Sample sizes for participants receiving GLP-1RAs ranged from 2,473 to 460,032, while the comparator groups included non-users of GLP-1RAs and users of other glucose-lowering therapies, such as metformin, insulin, sulfonylureas, dipeptidyl peptidase-4 inhibitors, and sodium–glucose cotransporter-2 inhibitors. The mean age of participants ranged from 51.5 to 74.0 years. Follow-up durations ranged from 660 days to 15 years, allowing for the assessment of both short- and long-term cancer outcomes.

**Table 2:** Characteristics of included GLP-1RAs cancer studies.

ID	Author(s)	Country	Study design	GLP-1RAs sample size	Comparator sample size	Mean age (years)	Follow-up duration
1	(14)	USA	Retrospective cohort	29,850	29,751	56.5	15 years
2	(15)	Israel	Observational retrospective cohort	3,178	3,178	52.3	Median 7.2–7.5 years; up to 12.9 years
3	(16)	USA	Retrospective cohort	3,162	3,162	57.3	5 years
4	(17)	USA	Retrospective PSM cohort	92,311	91,701	NR	15 years
5	(18)	USA	Retrospective target-trial emulation	41,112	310,801	65.3 ± 8.5	Median 660 days
6	(19)	USA	Retrospective PSM cohort	48,983	1,044,745	59.8 ± 15.1	Up to 15 years
7	(20)	USA	Retrospective PSM cohort	2,553–2,564	2,553–2,564	74.0	Median 1.65–1.73 years
8	(19)	USA	Retrospective cohort	48,983	1,044,745	59.8	Up to 15 years
9	(7)	USA	Retrospective cohort	21,362/20,962	21,362/20,962	72.8–73.3	Median 1.91–1.95 years
10	(21)	USA	Retrospective cohort (TriNetX)	245,532	Multiple matched cohorts	NR	15 years
11	(22)	UK	Population-based cohort study	2,473	NR	64.8	Mean 3.5 years
12	(23)	Multinational	Active-comparator new-user cohort	460,032	3,893,243	NR	NR
13	(24)	Multinational	Retrospective cohort study	112,735	112,735	57.5	5 years
14	(25)	USA	Retrospective multicenter cohort	146,277	146,277	58.3	7 years
15	(26)	Israel	Population-based historical cohort	33,377	106,849	59.9 ± 12.8	Mean 6.1 years; up to 9 years
16	(27)	Global	Target trial emulation cohort	28,934	28,934	65.6	5 years
17	(28)	France	Nested case-control study	NR	45,184 controls	NR	6 years
18	(5)	USA	Retrospective cohort	4,912	82,964	58.0	3 years
19	(29)	South Korea	Nationwide retrospective PSM cohort	2,609	2,609	51.5 ± 12.0	Median 8 years
20	(30)	USA	Retrospective cohort study	206,844	206,844	50.0 ± 13.9	5 years
21	(31)	USA	Retrospective cohort; target trial emulation	43,317	43,315	52.4 ± 14.5	2014–2024

GLP-1RAs, glucagon-like peptide-1 receptor agonists; PSM, propensity score matching; NR, not reported.

### 3.3 Overall cancer risk associated with GLP-1RAs

**Figure 2:**
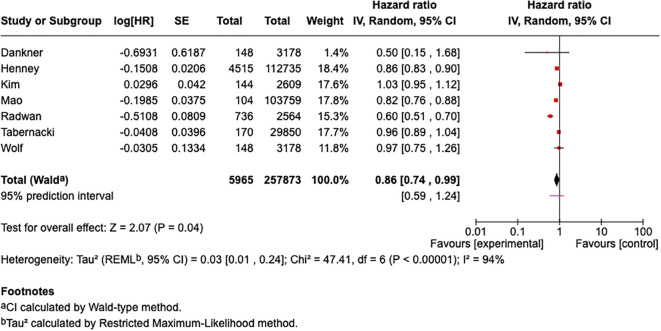
Forest plot showing the association between GLP-1RAs and overall cancer risk. The pooled hazard ratio indicated a significant reduction in overall cancer risk among GLP-1RAs users (HR = 0.86, 95% CI: 0.74–0.99; p = 0.04).

A total of seven studies involving 257,873 GLP-1RAs users and 5,965 cancer cases were included in the meta-analysis assessing the association between GLP-1RAs use and overall cancer risk. The pooled random-effects analysis demonstrated that GLP-1RAs use was associated with a significant 14% reduction in overall cancer risk compared with comparator groups (HR = 0.86, 95% CI: 0.74–0.99; p = 0.04) (**Figure 2**). Substantial heterogeneity was observed among the included studies (I² = 94%, χ² = 47.41, p < 0.00001), indicating considerable variability in effect estimates. Most studies reported effect estimates favoring a reduced overall cancer risk among GLP-1RAs users, although several studies reported no significant association. Despite the high heterogeneity, the overall pooled effect remained statistically significant (Z = 2.07, p = 0.04), supporting an overall protective association between GLP-1RAs use and cancer risk. However, the wide prediction interval (95% PI: 0.59–1.24) suggests that the magnitude and direction of the association may vary across different populations and settings.

### 3.4 Cancer-specific meta-analysis

#### 3.4.1 Thyroid cancer

**Figure 3:**
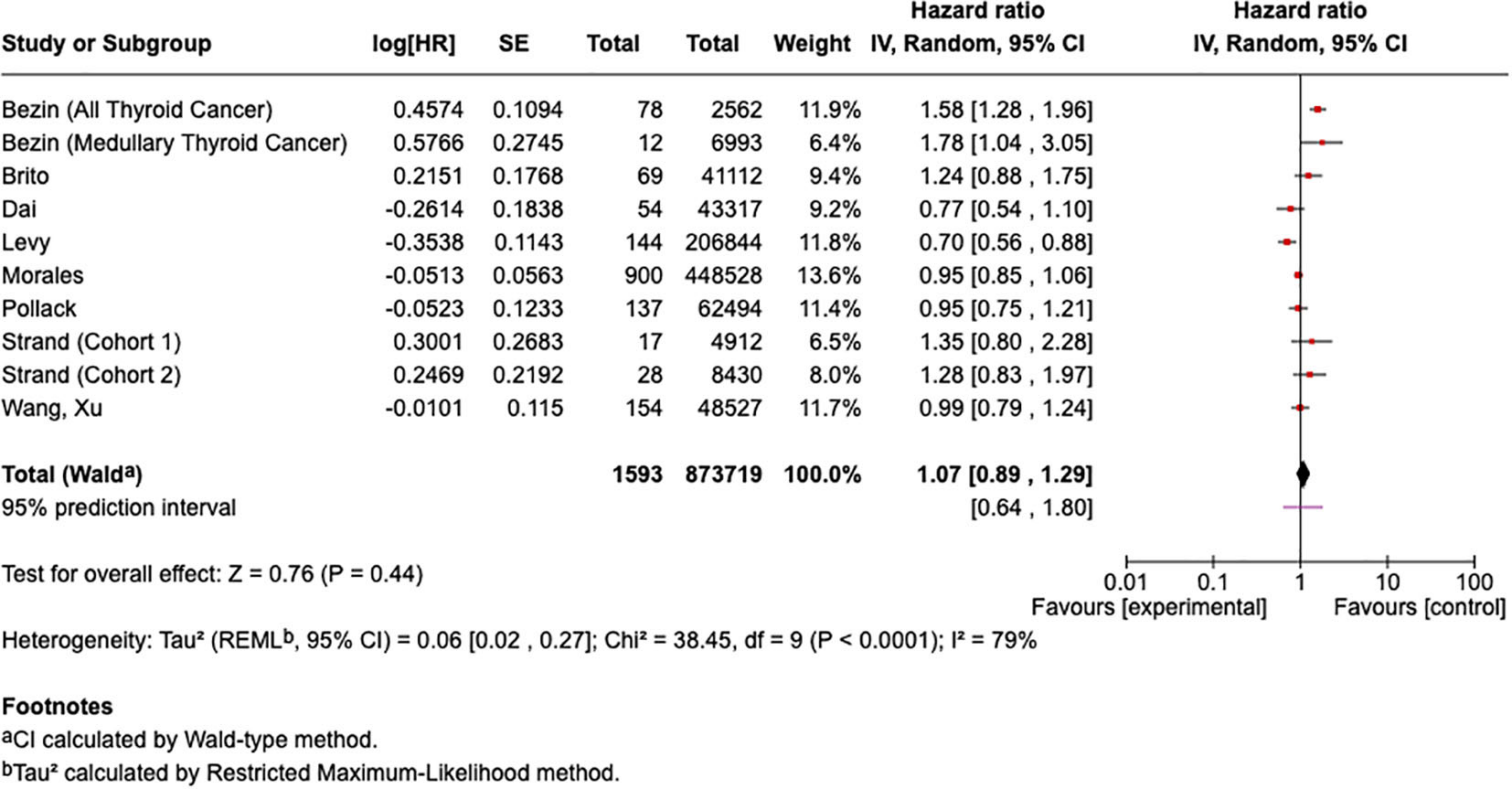
Association between GLP-1RAs use and thyroid cancer risk. Forest plot evaluating the association between GLP-1RAs use and thyroid cancer risk, showing no significant overall effect (HR = 1.07, 95% CI: 0.89–1.29; I² = 79%).

A total of 10 studies involving 873,719 participants were included in the meta-analysis assessing the association between GLP-1RAs use and thyroid cancer risk (**Table 3**). The pooled random-effects analysis demonstrated no significant association between GLP-1RAs use and thyroid cancer risk (HR = 1.07, 95% CI: 0.89–1.29; p = 0.44) (**Figure 3**). Substantial heterogeneity was observed among the included studies (I² = 79%, τ² = 0.06, p < 0.0001), indicating considerable variability in effect estimates. While some studies reported increased risks and others reported reduced risks, most effect estimates were close to the null value, resulting in an overall non-significant pooled effect. The prediction interval (95% PI: 0.64–1.80) suggests that future studies may report either a protective or harmful association.

**Table 3:** Summary of pooled meta-analysis estimates for the association between GLP-1 receptor agonist use and site-specific cancer risk.

Cancer type	No of studies	Participants	Pooled HR (95% CI)	p-value	I² (%)	Interpretation
Thyroid Cancer	10	873,719	1.07 (0.89–1.29)	0.44	79	No significant association
Breast Cancer	5	263,386	0.94 (0.78–1.13)	0.49	81	No significant association
Lung Cancer	4	301,373	0.71 (0.51–1.00)	0.05	91	Borderline risk reduction
Kidney Cancer	3	113,001	1.12 (0.74–1.71)	0.58	87	No significant association
Endometrial Cancer	4	272,895	0.76 (0.58–0.99)	0.04	78	Significant risk reduction
Pancreatic Cancer	8	495,678	0.65 (0.50–0.84)	0.001	86	Significant risk reduction
Colorectal Cancer	5	348,900	0.75 (0.59–0.96)	0.02	87	Significant risk reduction
Prostate Cancer	3	231,026	0.84 (0.67–1.05)	0.13	79	No significant association
Ovarian Cancer	3	261,962	0.57 (0.46–0.71)	<0.00001	0	Significant risk reduction
Hepatocellular Carcinoma	3	298,558	0.63 (0.44–0.88)	0.008	77	Significant risk reduction
Esophageal Cancer	3	401,558	0.48 (0.33–0.69)	<0.0001	70	Significant risk reduction
Gastric Cancer	3	401,570	0.54 (0.38–0.76)	0.0004	66	Significant risk reduction

#### 3.4.2 Breast cancer

**Figure 4:**
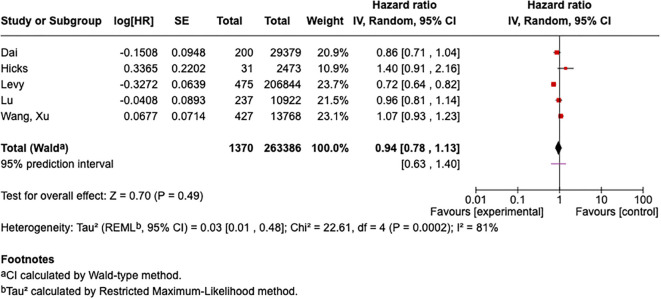
Association between GLP-1RAs use and breast cancer risk. Forest plot showing the pooled association between GLP-1RAs use and breast cancer risk. The random-effects meta-analysis demonstrated no significant association between GLP-1RAs use and breast cancer risk (HR = 0.94, 95% CI: 0.78–1.13; I² = 81%).

A total of five studies involving 263,386 participants and 1,370 breast cancer cases among GLP-1RAs users were included in the meta-analysis assessing the association between GLP-1RAs use and breast cancer risk (**Table 3**). The pooled random-effects analysis demonstrated no significant association between GLP-1RAs use and breast cancer risk (HR = 0.94, 95% CI: 0.78–1.13; p = 0.49) (**Figure 4**). Substantial heterogeneity was observed across the included studies (I² = 81%, τ² = 0.03, p = 0.0002). Most studies reported effect estimates close to the null value, although some studies suggested either a protective association or an increased risk. The prediction interval (95% PI: 0.63–1.40) suggests uncertainty regarding the direction and magnitude of the association that may be observed in future studies.

#### 3.4.3 Lung cancer

**Figure 5:**
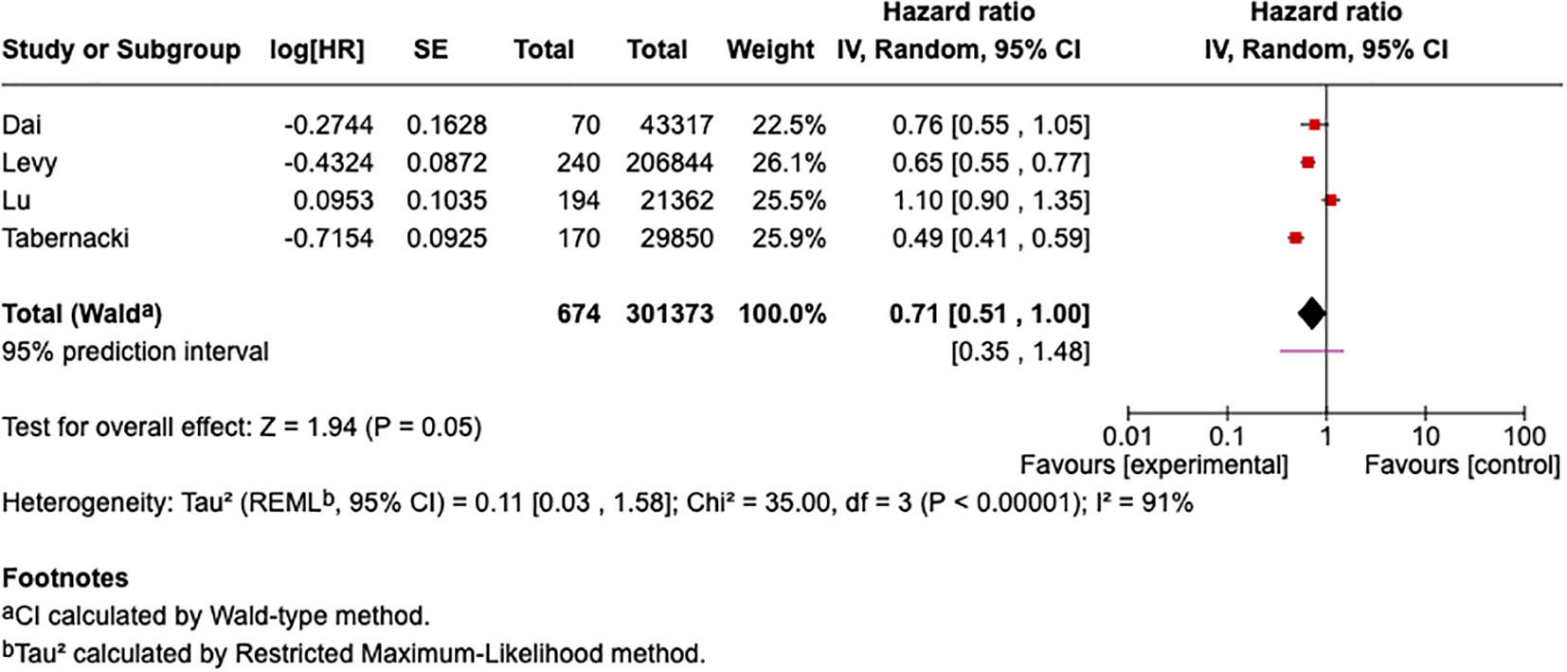
Association between GLP-1RAs use and lung cancer risk. Forest plot of 4 studies evaluating the association between GLP-1RAs use and lung cancer risk, showing a borderline reduction in risk (HR = 0.71, 95% CI: 0.51–1.00; I² = 91%).

A total of four studies involving 301,373 participants were included in the meta-analysis assessing the association between GLP-1RAs use and lung cancer risk (**Table 3**). The pooled random-effects analysis suggested a borderline reduction in lung cancer risk, although the association did not reach conventional statistical significance (HR = 0.71, 95% CI: 0.51–1.00; p = 0.05) (**Figure 5**). Considerable heterogeneity was observed among the included studies (I² = 91%, τ² = 0.11, p < 0.00001). Most studies reported effect estimates favoring a reduced risk of lung cancer among GLP-1RAs users, although one study reported no significant association. The prediction interval (95% PI: 0.35–1.48) suggests uncertainty regarding the magnitude and direction of the association that may be observed in future studies.

#### 3.4.4 Kidney cancer

**Figure 6:**
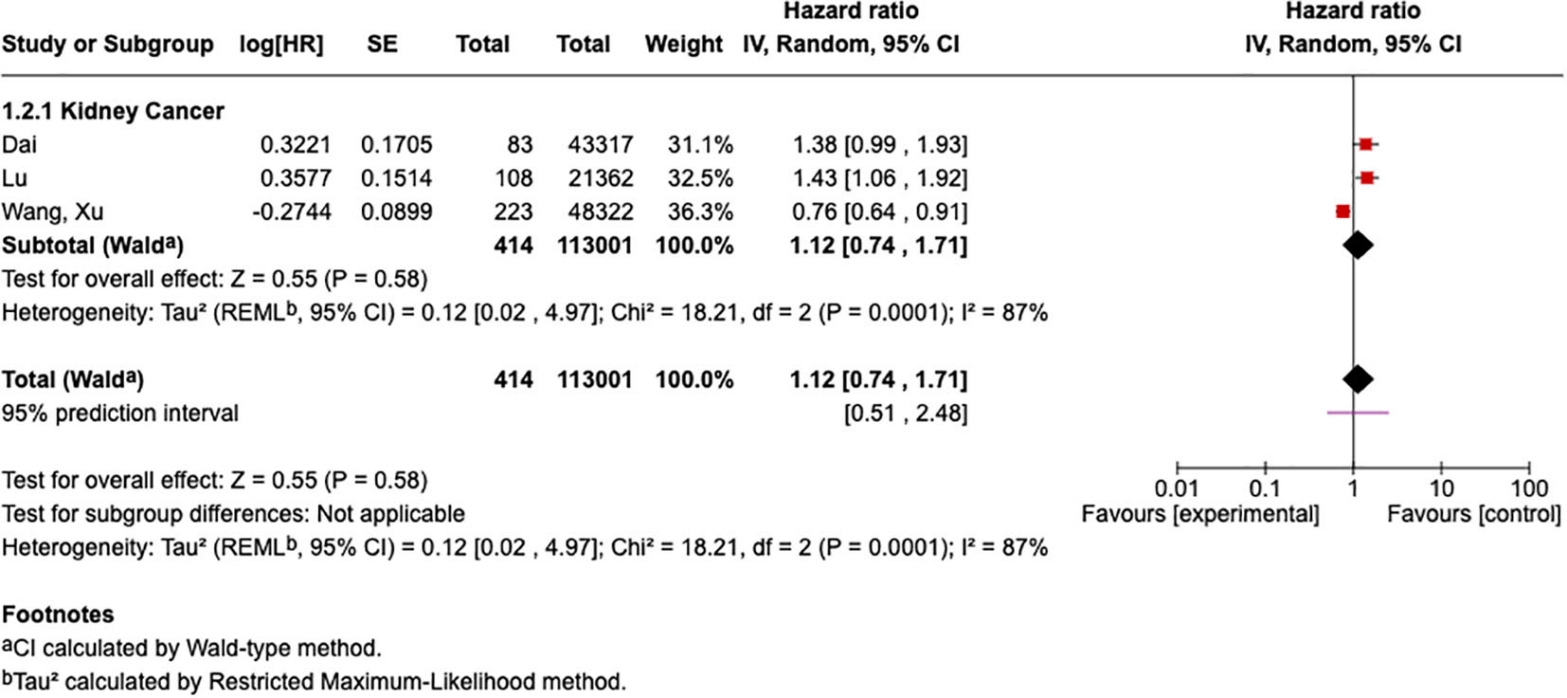
Association between GLP-1RAs use and kidney cancer risk. Forest plot evaluating the association between GLP-1RAs use and kidney cancer risk, showing no significant overall effect (HR = 1.12, 95% CI: 0.74–1.71; I² = 87%).

A total of three studies involving 113,001 participants were included in the meta-analysis assessing the association between GLP-1RAs use and kidney cancer risk (**Table 3**). The pooled random-effects analysis showed no significant association between GLP-1RAs use and kidney cancer risk (HR = 1.12, 95% CI: 0.74–1.71; p = 0.58) (**Figure 6**). Substantial heterogeneity was observed among the included studies (I² = 87%, τ² = 0.12, p = 0.0001). While some studies reported an increased risk of kidney cancer among GLP-1RAs users, others reported a reduced risk, resulting in an overall non-significant pooled effect. The prediction interval (95% PI: 0.51–2.48) suggests substantial uncertainty regarding the magnitude and direction of the association that may be observed in future studies.

#### 3.4.5 Endometrial cancer

**Figure 7:**
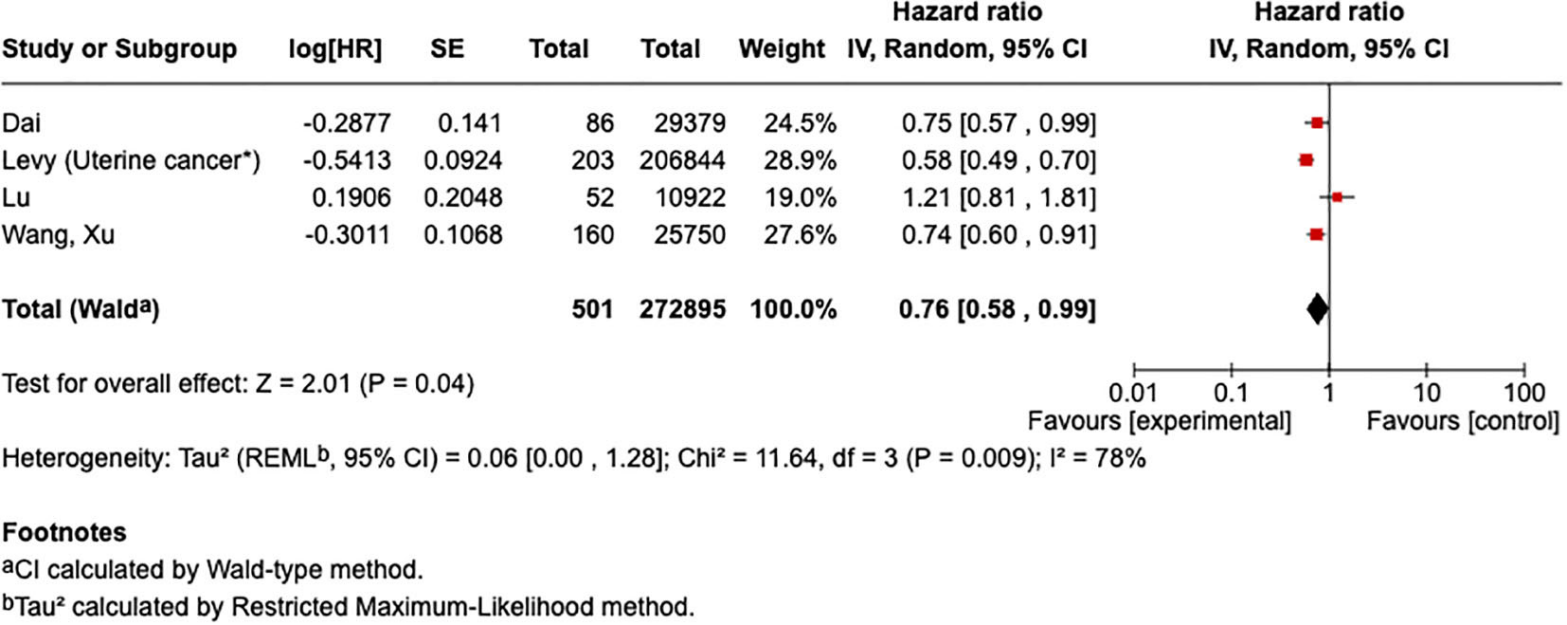
Association between GLP-1RAs use and endometrial cancer risk. Forest plot evaluating the association between GLP-1RAs use and endometrial cancer risk, showing a significant reduction in risk among GLP-1RAs users (HR = 0.76, 95% CI: 0.58–0.99; I² = 78%).

A total of four studies involving 272,895 participants were included in the meta-analysis assessing the association between GLP-1RAs use and endometrial cancer risk (**Table 3**). The pooled random-effects analysis demonstrated a significant reduction in endometrial cancer risk among GLP-1RAs users compared with comparator groups (HR = 0.76, 95% CI: 0.58–0.99; p = 0.04) (**Figure 7**). Substantial heterogeneity was observed across the included studies (I² = 78%, τ² = 0.06, p = 0.009). Most studies reported effect estimates favoring a reduced risk of endometrial cancer among GLP-1RAs users, although one study reported a non-significant increase in risk.

#### 3.4.6 Pancreatic cancer

**Figure 8:**
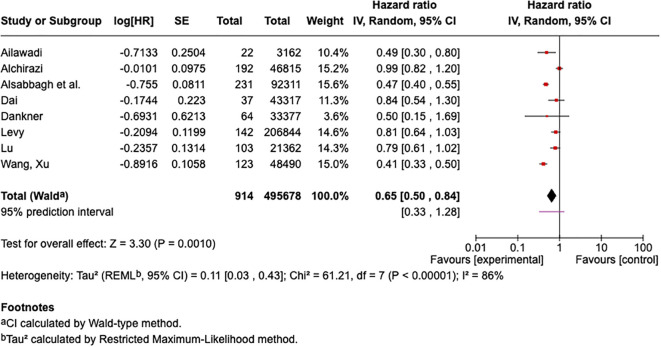
Association between GLP-1RAs use and pancreatic cancer risk. Forest plot evaluating the association between GLP-1RAs use and pancreatic cancer risk, showing a significant reduction in risk among GLP-1RAs users (HR = 0.65, 95% CI: 0.50–0.84; I² = 86%).

A total of eight studies involving 495,678 participants were included in the meta-analysis assessing the association between GLP-1RAs use and pancreatic cancer risk (**Table 3**). The pooled random-effects analysis demonstrated a significant reduction in pancreatic cancer risk among GLP-1RAs users compared with comparator groups (HR = 0.65, 95% CI: 0.50–0.84; p = 0.001) (**Figure 8**). Considerable heterogeneity was observed across the included studies (I² = 86%, τ² = 0.11, p < 0.00001). Most studies reported effect estimates favoring a reduced risk of pancreatic cancer among GLP-1RAs users, although the magnitude of the association varied across studies. A small number of studies reported neutral or non-significant associations. The prediction interval (95% PI: 0.33–1.28) suggests that future studies may observe either a substantial protective effect or no significant association.

#### 3.4.7 Colorectal cancer

**Figure 9:**
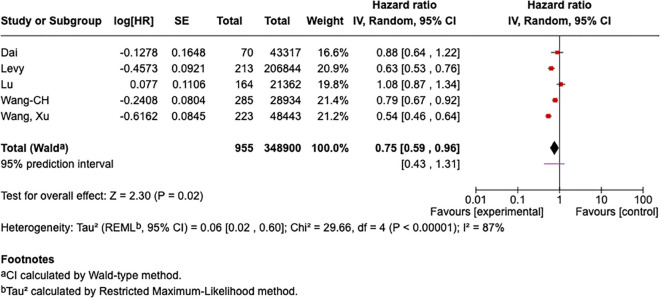
Association between GLP-1RAs use and colorectal cancer risk. Forest plot evaluating the association between GLP-1RAs use and colorectal cancer risk, showing a significant reduction in risk among GLP-1RAs users (HR = 0.75, 95% CI: 0.59–0.96; I² = 87%).

A total of five studies involving 348,900 participants were included in the meta-analysis assessing the association between GLP-1RAs use and colorectal cancer risk (**Table 3**). The pooled random-effects analysis demonstrated a significant reduction in colorectal cancer risk among GLP-1RAs users compared with comparator groups (HR = 0.75, 95% CI: 0.59–0.96; p = 0.02) (**Figure 9**). Substantial heterogeneity was observed across the included studies (I² = 87%, τ² = 0.06, p < 0.00001). Most studies reported effect estimates favoring a reduced risk of colorectal cancer among GLP-1RAs users, although the magnitude of the association varied across studies. Some studies reported non-significant increases or reductions in risk. The prediction interval (95% PI: 0.43–1.31) suggests variability in the effect that may be observed in future studies, ranging from a protective association to no significant effect.

#### 3.4.8 Prostate cancer

**Figure 10:**
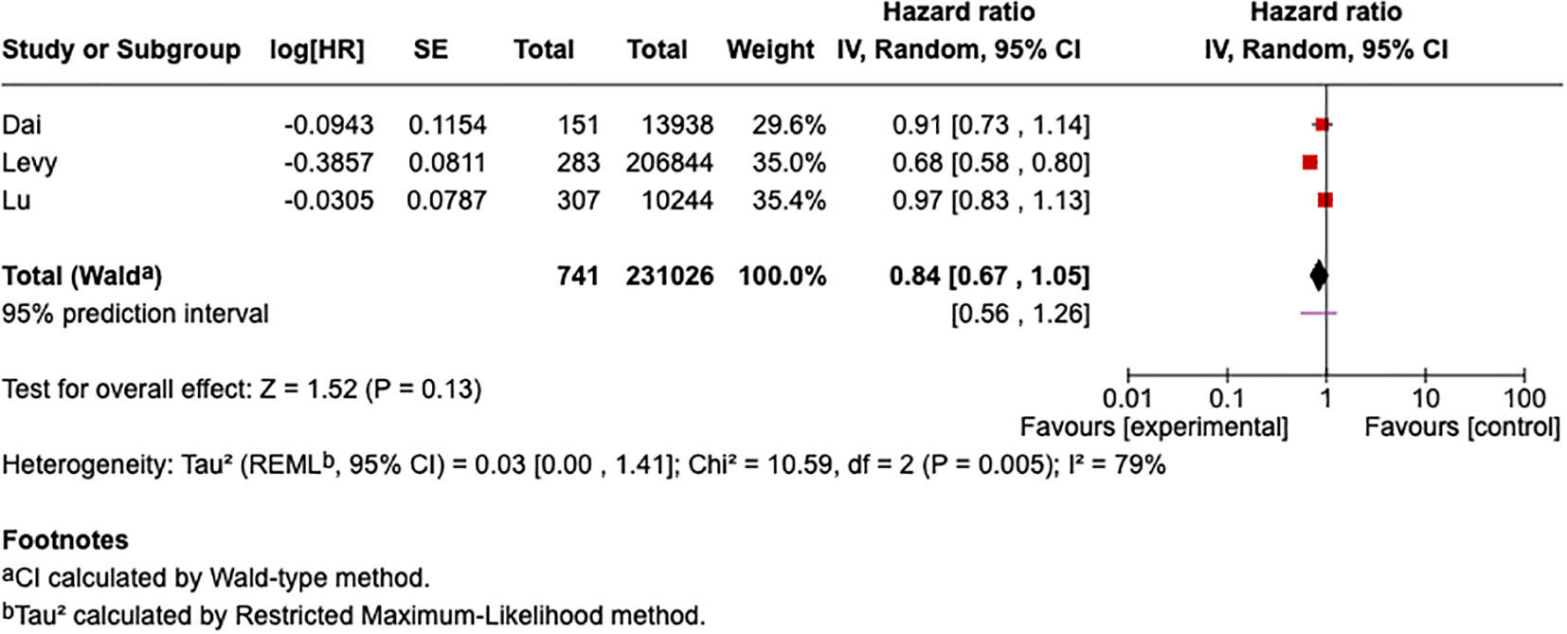
Association between GLP-1RAs use and prostate cancer risk. Forest plot evaluating the association between GLP-1RAs use and prostate cancer risk, showing no significant overall effect (HR = 0.84, 95% CI: 0.67–1.05; I² = 79%).

A total of three studies involving 231,026 participants were included in the meta-analysis assessing the association between GLP-1RAs use and prostate cancer risk (**Table 3**). The pooled random-effects analysis showed no significant association between GLP-1RAs use and prostate cancer risk (HR = 0.84, 95% CI: 0.67–1.05; p = 0.13) (**Figure 10**). Substantial heterogeneity was observed among the included studies (I² = 79%, τ² = 0.03, p = 0.005). Most studies reported effect estimates favoring a reduced risk of prostate cancer among GLP-1RAs users, although the associations were generally not statistically significant. The prediction interval (95% PI: 0.56–1.26) suggests that future studies may report effects ranging from a modest protective association to no effect.

#### 3.4.9 Ovarian cancer

**Figure 11:**
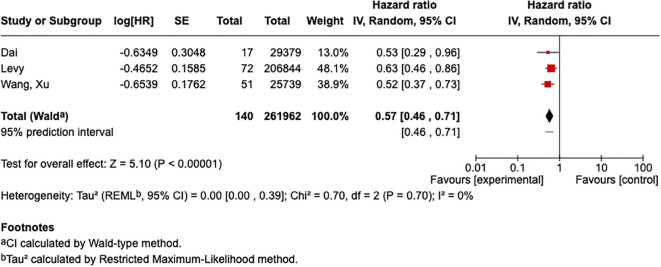
Association between GLP-1RAs use and ovarian cancer risk. Forest plot evaluating the association between GLP-1RAs use and ovarian cancer risk, showing a significant reduction in risk among GLP-1RAs users (HR = 0.57, 95% CI: 0.46–0.71; I² = 0%).

A total of three studies involving 261,962 participants were included in the meta-analysis assessing the association between GLP-1RAs use and ovarian cancer risk (**Table 3**). The pooled random-effects analysis demonstrated a significant reduction in ovarian cancer risk among GLP-1RAs users compared with comparator groups (HR = 0.57, 95% CI: 0.46–0.71; p < 0.00001) (**Figure 11**). No significant heterogeneity was observed among the included studies (I² = 0%, τ² = 0.00, p = 0.70). All included studies reported effect estimates favoring a reduced risk of ovarian cancer among GLP-1RAs users. The prediction interval (95% PI: 0.46–0.71) remained entirely below the null value.

#### 3.4.10 Hepatocellular carcinoma (liver cancer)

**Figure 12:**
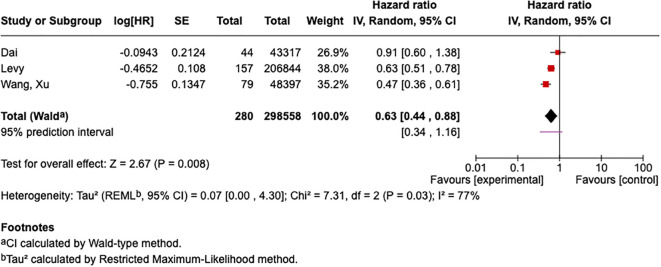
Association between GLP-1RAs use and hepatocellular carcinoma risk. Forest plot evaluating the association between GLP-1RAs use and hepatocellular carcinoma risk, showing a significant reduction in risk among GLP-1RAs users (HR = 0.63, 95% CI: 0.44–0.88; I² = 77%).

A total of three studies involving 298,558 participants were included in the meta-analysis assessing the association between GLP-1RAs use and hepatocellular carcinoma risk (**Table 3**). The pooled random-effects analysis demonstrated a significant reduction in hepatocellular carcinoma risk among GLP-1RAs users compared with comparator groups (HR = 0.63, 95% CI: 0.44–0.88; p = 0.008) (**Figure 12**). Substantial heterogeneity was observed among the included studies (I² = 77%, τ² = 0.07, p = 0.03). Most studies reported effect estimates favoring a reduced risk of hepatocellular carcinoma among GLP-1RAs users, although the magnitude of the association varied across studies. The prediction interval (95% PI: 0.34–1.16) suggests that future studies may observe effects ranging from a reduction in risk to little or no association.

#### 3.4.11 Esophageal cancer

**Figure 13:**
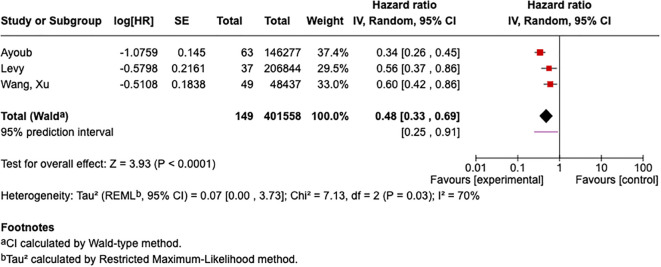
Association between GLP-1RAs use and esophageal cancer risk. Forest plot evaluating the association between GLP-1RAs use and esophageal cancer risk, showing a significant reduction in risk among GLP-1RAs users (HR = 0.48, 95% CI: 0.33–0.69; I² = 70%).

A total of three studies involving 401,558 participants were included in the meta-analysis assessing the association between GLP-1RAs use and esophageal cancer risk (**Table 3**). The pooled random-effects analysis demonstrated a significant reduction in esophageal cancer risk among GLP-1RAs users compared with comparator groups (HR = 0.48, 95% CI: 0.33–0.69; p < 0.0001) (**Figure 13**). Moderate to substantial heterogeneity was observed among the included studies (I² = 70%, τ² = 0.07, p = 0.03). Nevertheless, all included studies reported effect estimates favoring a reduced risk of esophageal cancer among GLP-1RAs users. The prediction interval (95% PI: 0.25–0.91) remained below the null value, suggesting that future studies are likely to observe a protective association.

#### 3.4.12 Gastric (stomach) cancer

**Figure 14:**
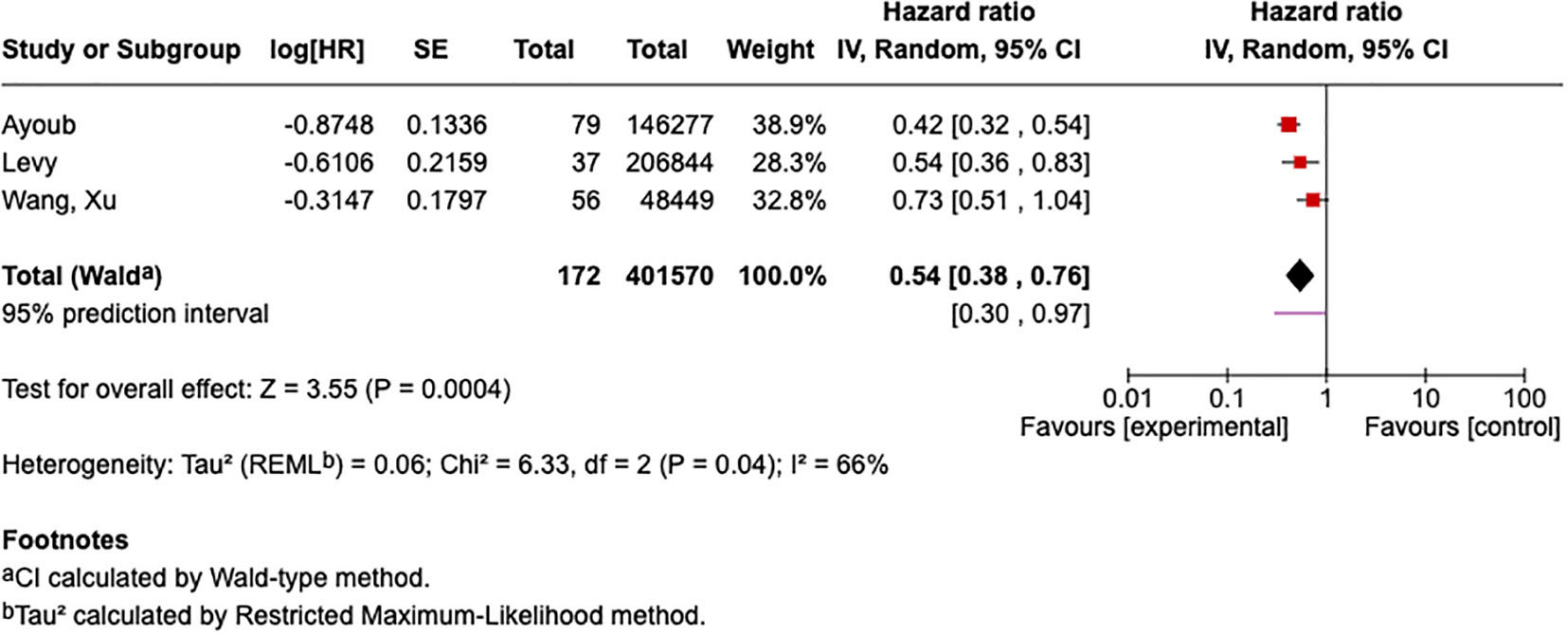
Association between GLP-1RAs use and gastric cancer risk. Forest plot evaluating the association between GLP-1RAs use and gastric cancer risk, showing a significant reduction in risk among GLP-1RAs users (HR = 0.54, 95% CI: 0.38–0.76; I² = 66%).

A total of three studies involving 401,570 participants were included in the meta-analysis assessing the association between GLP-1RAs use and gastric (stomach) cancer risk (**Table 3**). The pooled random-effects analysis demonstrated a significant reduction in gastric cancer risk among GLP-1RAs users compared with comparator groups (HR = 0.54, 95% CI: 0.38–0.76; p = 0.0004) (**Figure 14**). Moderate heterogeneity was observed among the included studies (I² = 66%, τ² = 0.06, p = 0.04). Most studies reported effect estimates favoring a reduced risk of gastric cancer among GLP-1RAs users, although the magnitude of the association varied between studies. The prediction interval (95% PI: 0.30–0.97) remained below the null value.

### 3.5 Narrative synthesis of cancer outcomes with insufficient studies for meta-analysis

Four cancer outcomes (gallbladder cancer, bladder cancer, multiple myeloma, and meningioma) were reported by fewer than three studies and were therefore not eligible for quantitative meta-analysis (**Table 4**). For gallbladder cancer, a single study reported a significantly reduced risk among GLP-1RAs users (HR = 0.35, 95% CI: 0.15–0.83). For bladder cancer, two studies found no significant association between GLP-1RAs use and cancer risk (HRs ranging from 0.97 to 1.08). For multiple myeloma, both available studies suggested a reduced risk among GLP-1RAs users (HRs: 0.59–0.79). Similarly, for meningioma, both studies reported protective associations, with hazard ratios ranging from 0.37 to 0.69.

**Table 4:** Summary of cancer outcomes with insufficient studies for meta-analysis.

Cancer type	No. of studies	Participants (GLP-1RAs/comparator)	Key findings	Interpretation	Reference(s)
Gallbladder Cancer	1	48,587/48,587	Significantly reduced risk (HR = 0.35, 95% CI: 0.15–0.83).	Potential protective effect	(19)
Bladder Cancer	2	64,679/64,677	No significant association across available studies (HRs 0.97 and 1.08).	No clear association between GLP-1RAs use and bladder cancer risk.	(20, 31)
Multiple Myeloma	2	91,844/91,842	Protective associations reported (HRs 0.59 and 0.79).	Possible risk reduction	(19, 31)
Meningioma	2	91,835/91,833	Both studies reported significant reductions in risk (HRs 0.37 and 0.69).	Preliminary evidence suggests reduced risk among GLP-1RAs users.	(19, 31)

CI, confidence interval; GLP-1RAs, glucagon-like peptide-1 receptor agonists; HR, Hazard ratios.

### 3.6 Biological mechanisms linking GLP-1RAs and cancer

Mechanistic evidence linking GLP-1RAs to cancer outcomes was limited (**Table 5**). Most included clinical studies evaluated epidemiological associations and did not directly assess molecular or biomarker-based mechanisms. Therefore, the biological pathways underlying the observed cancer associations remain uncertain. Available mechanistic evidence suggested that GLP-1RAs may influence cancer-related processes through modulation of metabolic regulation, inflammation, cellular proliferation, apoptosis, and intracellular signaling pathways. Reported pathways included PI3K/AKT, MAPK/ERK, mTOR, and apoptosis-related markers such as Bax, Bcl-2, and Ki-67. However, these findings were reported in only a small number of studies and should be interpreted cautiously.

**Table 5:** Cancer-related biological pathways potentially influenced by GLP-1RAs.

Mechanistic pathway	GLP-1RAs reported	Reported findings	Reference(s)
PI3K/AKT Pathway	Semaglutide, Liraglutide	Reduced tumor cell growth, survival, and proliferation	(16, 24, 30)
MAPK/ERK Pathway	Semaglutide, Liraglutide	Reduced cellular proliferation and tumor progression	(16, 19, 30, 32)
mTOR Pathway	Semaglutide, Tirzepatide	Reduced cancer cell growth and survival	(16, 32)
NF-κB Pathway	Semaglutide, Tirzepatide	Reduced inflammation-driven carcinogenesis	(16, 24)
IGF-1 Signaling Pathway	Semaglutide, Tirzepatide	Reduced cellular proliferation and oncogenic signaling	(32)
Wnt/β-Catenin Pathway	Semaglutide, Tirzepatide	Reduced colorectal tumor development and progression	(32)
Apoptotic Pathways	Semaglutide, Liraglutide, Tirzepatide	Increased elimination of malignant cells	(8, 16)
Cell Proliferation Pathways	Semaglutide, Liraglutide	Reduced tumor growth and progression	(24, 31)
Thyroid C-cell Signaling (Hypothesized)	GLP-1RAs class	Proposed mechanism for thyroid tumorigenesis; evidence remains inconsistent	(23, 28, 33)

### 3.7 Risk of bias assessment

**Figure 15:**
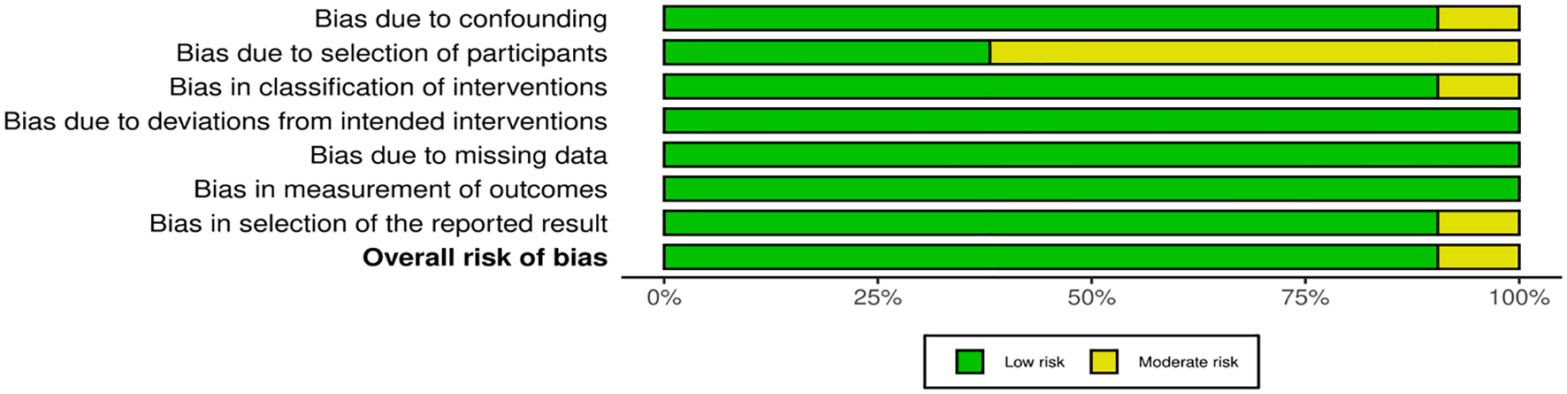
ROBINS-I risk of bias summary plot across all included studies. Studies were at low to moderate risk of bias, with a smaller proportion showing serious concerns primarily in the confounding and participant selection domains.

**Figure 16:**
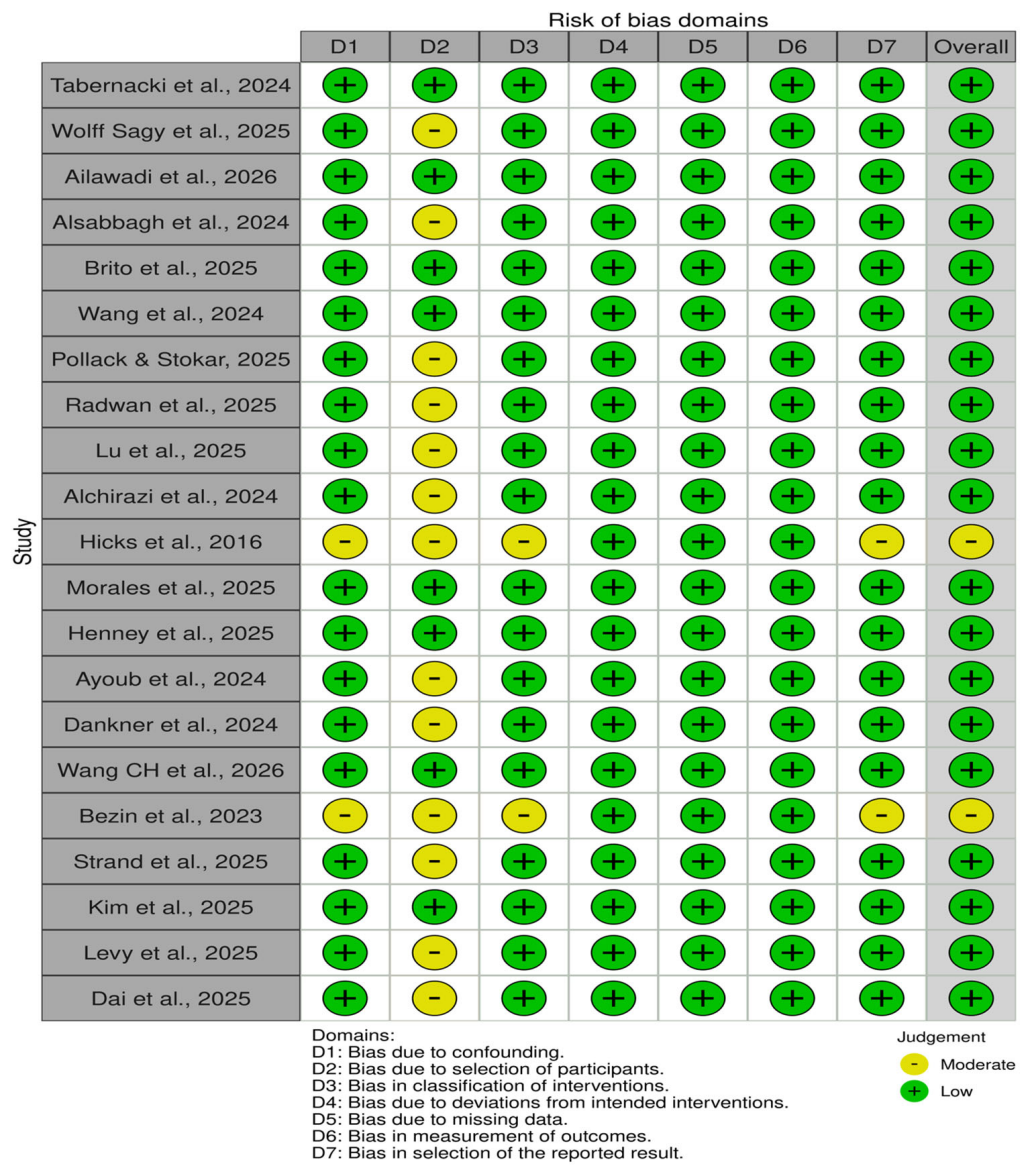
Risk of bias assessment across the seven ROBINS-I domains for all included studies.

The risk of bias across the included studies was assessed across seven domains: confounding (D1), participant selection (D2), classification of interventions (D3), deviations from intended interventions (D4), missing data (D5), measurement of outcomes (D6), and selection of the reported result (D7). Each study was assigned a judgment of low, moderate, or serious risk for each domain, and an overall risk of bias rating was determined based on the highest level of risk identified across domains. Most studies were rated as having low or moderate risk of bias across the assessed domains. Moderate-risk ratings were most observed in the domains of confounding and participant selection. The proportion of studies classified as low, moderate, or serious risk of bias across each domain was also summarized (**Figures 15**, **16**).

## 4 Discussion

The pooled analysis demonstrated a significant reduction in overall cancer risk among GLP-1RAs users (HR = 0.86, 95% CI: 0.74–0.99; p = 0.04), with significant protective associations observed for pancreatic, colorectal, endometrial, ovarian, hepatocellular, esophageal, and gastric cancers. In contrast, no significant association was observed for thyroid, breast, kidney, and prostate cancers. These findings are consistent with recent systematic reviews and meta-analyses reporting no convincing evidence that GLP-1RAs increase overall cancer risk (34). Recent randomized-trial-based meta-analyses have concluded that GLP-1RAs have little or no effect on most cancer outcomes, including thyroid, pancreatic, breast, and kidney cancers (35). Our findings extend this evidence by incorporating large real-world cohort studies with longer follow-up periods and by examining site-specific cancer outcomes separately.

For thyroid cancer, the pooled estimate showed no significant association, although heterogeneity was substantial. This aligns with recent evidence suggesting that GLP-1RAs may not clearly be associated with increased thyroid cancer risk in human populations (36). The variability observed across studies may be due to differences in comparator groups, follow-up duration, detection bias, and outcome definitions, particularly where studies combined all thyroid cancers with medullary thyroid cancer (28). For gastrointestinal cancers, our findings suggested significant reductions in pancreatic, colorectal, hepatocellular, esophageal, and gastric cancer risks. These findings are consistent with previous meta-analyses suggesting that GLP-1RAs may reduce the risk of obesity-related cancers through weight loss, improved insulin sensitivity, reduced chronic inflammation, and modulation of metabolic pathways involved in carcinogenesis (35). Our findings are contrary to a recent meta-analysis that reported an increased risk of colorectal cancer among GLP-1RA users (RR = 2.31, 95% CI: 1.82–2.93), although no significant difference in colorectal cancer incidence was observed when GLP-1RA users were compared with users of other glucose-lowering therapies (37). However, the high heterogeneity observed for several gastrointestinal cancer outcomes may suggest that the magnitude of benefit may differ across populations and comparator groups. Our finding of a reduced risk of hepatocellular carcinoma among GLP-1RA users is consistent with that of Dalbeni et al. (38), who reported a 42% lower hepatocellular carcinoma risk (HR = 0.60, 95% CI: 0.41–0.88). Their meta-regression identified comparator drug class, particularly insulin, as a major source of heterogeneity, whereas our analysis included only three studies and showed substantial heterogeneity (I² = 77%), limiting further exploration of heterogeneity sources. Although significant reductions were observed for colorectal and pancreatic cancers, and a borderline reduction was observed for endometrial and lung cancers, the corresponding prediction intervals crossed the null value. Together with the substantial heterogeneity observed across studies, these findings suggest that future studies may report weaker associations or no effect. Therefore, these results should be interpreted cautiously and considered suggestive, requiring confirmation in larger prospective studies.

Furthermore, the significant reduction in ovarian and endometrial cancer risk is biologically plausible, given the relationship between obesity, insulin resistance, chronic inflammation, and hormone-sensitive cancers (39). These findings are consistent with recent observational studies reporting lower risks of gynecological cancers among GLP-1RAs users (40). Nevertheless, causal interpretation remains limited because most included studies were observational. In contrast, no significant association was observed for breast, kidney, or prostate cancers. For kidney cancer, the direction of effect varied across studies, with some estimates suggesting increased risk and others suggesting reduced risk. This inconsistency may be explained by differences in baseline kidney disease, diabetes severity, comparator selection, and residual confounding. Therefore, findings on kidney cancer should be interpreted cautiously, since the number of studies was limited.

Mechanistic evidence supporting the association between GLP-1RAs and cancer risk was limited. A small number of studies suggested that GLP-1RAs may influence biological processes involved in tumor development, including chronic inflammation, cellular proliferation, apoptosis, and metabolic regulation. Experimental studies reported modulation of signaling pathways such as PI3K/AKT, MAPK/ERK, and mTOR, which are known to play important roles in cancer initiation and progression. However, most studies included in this review were observational and did not directly investigate these molecular mechanisms. Consequently, although the observed reductions in cancer risk are biologically plausible, the current evidence is insufficient to determine whether these associations result from direct anti-tumor effects of GLP-1RAs or from indirect effects related to weight loss, improved glycemic control, and reduced systemic inflammation.

The distribution of GLP-1RAs across the included studies should also be considered when interpreting the findings. Liraglutide and semaglutide were the most frequently investigated agents, whereas evidence for lixisenatide, tirzepatide, and albiglutide was limited. Consequently, the pooled estimates are driven by data from liraglutide- and semaglutide-based studies and may not be fully generalizable to all GLP-1RAs. In particular, evidence for tirzepatide remains limited, and its findings should be interpreted cautiously because its mechanism of action involves activation of both GIP and GLP-1 receptors.

## 5 Limitations

Despite the strength of the current studies, there are some limitations that must be acknowledged. First, substantial heterogeneity was observed across several cancer-specific analyses due to differences in populations, comparators, follow-up durations, and outcome definitions. Second, several studies had short follow-up periods, which may be insufficient to capture cancers with long latency periods. Third, **s**everal cancer outcomes were represented by a few studies, reducing the precision of the pooled estimates. Fourth, most studies reported epidemiological associations without direct molecular or biomarker validation, limiting mechanistic interpretation. Fifth, evidence was mostly driven by liraglutide and semaglutide, while data for other agents, particularly tirzepatide, were limited. Sixth, insufficient evidence was available for lymphoma, leukemia, and other hematological cancers, precluding meaningful conclusions. Seventh, no eligible studies were identified between 2017 and 2022 despite the growth in GLP-1RA research during this period. This gap may be attributed to the eligibility criteria requiring studies to report cancer-related outcomes and extractable effect estimates among adults with T2DM exposed to GLP-1Ras, as many studies published during this period focused on metabolic or cardiovascular outcomes rather than cancer outcomes. Eighth, most studies were conducted in high-income countries, which may limit the applicability of the findings to other settings. Nineth, multiple cancer-specific analyses were performed without adjustment for multiple comparisons, even though some studies were derived from overlapping database sources, potentially introducing non-independence of effect estimates.

## 6 Conclusion

This systematic review and meta-analysis found that the use of GLP-1RAs is associated with a significant reduction in overall cancer risk and several site-specific cancers, including pancreatic, colorectal, endometrial, ovarian, hepatocellular, esophageal, and gastric cancers. No significant associations were observed for thyroid, breast, kidney, or prostate cancers. However, this finding should be interpreted with caution for some cancer types represented by a few studies and some cancer types (e.g., colorectal, pancreatic, and lung) whose prediction intervals crossed the null, as future studies may plausibly show no effect, consistent with the high I² reported for them. Mechanistic evidence was limited but suggested that GLP-1RAs may influence pathways involved in inflammation, cellular proliferation, apoptosis, and metabolic regulation. However, most evidence was derived from observational studies, and direct molecular validation remains scarce. Consequently, the biological mechanisms underlying the observed associations are not yet fully understood. Although these findings support the overall oncologic safety of GLP-1RAs and suggest potential protective effects against certain cancers, substantial heterogeneity was observed across several analyses. Furthermore, evidence for some GLP-1RAs, particularly newer agents such as tirzepatide, remains limited. Future large-scale prospective studies, randomized trials with long-term follow-up, and mechanistic investigations are needed to clarify causality, determine drug-specific effects, and better understand the biological pathways linking GLP-1RAs to cancer outcomes.

## Data Availability

The original contributions presented in the study are included in the article/**Supplementary Material**. Further inquiries can be directed to the corresponding authors.

## References

[1] Andreadis P Karagiannis T Malandris K Avgerinos I Liakos A Manolopoulos A . Semaglutide for type 2 diabetes mellitus: a systematic review and meta-analysis. Diabetes Obes Metab. (2018) 20:2255–63. doi: 10.1111/DOM.13361 29756388

[2] WHO . Analytical Fact Sheet Fact Sheet Diabetes, a silent killer in Africa. (2024).

[3] Hossain MJ Al-Mamun M Islam MR . Diabetes mellitus, the fastest growing global public health concern: early detection should be focused. Health Sci Rep. (2024) 7:e2004. doi: 10.1002/HSR2.2004 38524769 PMC10958528

[4] Zhang Y Xie Y Xia S Ge X Li J Liu F . The novel dual GIP and GLP-1 receptor agonist tirzepatide attenuates colon cancer development by regulating glucose metabolism. Adv Sci. (2025) 12:2411980. doi: 10.1002/ADVS.202411980 40125821 PMC12097124

[5] Strand MW Chow D Shen W Watanabe JH . Impact of incretin mimetics on thyroid cancer among patients with type 2 diabetes: a retrospective cohort time-to-event analysis. Pharmacoepidemiology. (2025) 4:9. doi: 10.3390/PHARMA4020009 30654563

[6] Wang L Xu R Kaelber DC Berger NA . Glucagon-like peptide 1 receptor agonists and 13 obesity-associated cancers in patients with type 2 diabetes. JAMA Netw Open. (2024) 7:e2421305. doi: 10.1001/JAMANETWORKOPEN.2024.21305 38967919 PMC11227080

[7] Lu Y Dai H Tang H Donahoo WT George TJ Sun RC . Association of glucagon-like peptide-1 receptor agonists with cancer risk in older adults with type 2 diabetes. Obesity. (2025), 1–13. doi: 10.1002/OBY.24366 40841345 PMC12906048

[8] Mao X Zhang X Lai R Cheung KS Yuen MF Cheung R . Glucagon-like peptide 1 receptor agonist and reduced liver and non-liver complications in adults with type 2 diabetes and metabolic dysfunction-associated steatotic liver disease: a target trial emulation study. Clin Mol Hepatol. (2025) 31:1084. doi: 10.3350/CMH.2024.1096 40268291 PMC12260620

[9] Barać M Roganović J . GLP-1 receptor signaling and oral dysfunction: a narrative review on the mechanistic basis of semaglutide-related oral adverse effects. Biology. (2025) 14. doi: 10.3390/BIOLOGY14121650 41463424 PMC12729639

[10] Zhang J Androutsakos T Zhang F Yang L Lu X . Glucagon-like peptide-1 and dual/triple receptor agonists in the treatment of metabolic dysfunction-associated steatotic liver disease: advances in mechanistic research. Front Med (Lausanne). (2026) 13:1763185. doi: 10.3389/fmed.2026.1763185 41767516 PMC12935680

[11] Pollack R Stokar J . Long‐term glucagon‐like peptide 1 receptor agonist use is not associated with increased risk of thyroid cancer in adults with type 2 diabetes. Diabetes Metab Res Rev. (2025) 41. doi: 10.1002/dmrr.70104 41182904 PMC12582397

[12] Silverii GA Marinelli C Bettarini C Del Vescovo GG Monami M Mannucci E . GLP-1 receptor agonists and the risk for cancer: a meta-analysis of randomized controlled trials. Diabetes Obes Metab. (2025) 27:4454–68. doi: 10.1111/dom.16489 40437949 PMC12232360

[13] Page MJ McKenzie JE Bossuyt PM Boutron I Hoffmann TC Mulrow CD . The PRISMA 2020 statement: an updated guideline for reporting systematic reviews. BMJ. (2021):n71. doi: 10.1136/bmj.n71 33782057 PMC8005924

[14] Tabernacki T Wang L Kaelber DC Xu R Berger NA . Non-insulin antidiabetic agents and lung cancer risk in drug-naive patients with type 2 diabetes mellitus: a nationwide retrospective cohort study. Cancers (Bsl). (2024) 16. doi: 10.3390/CANCERS16132377 39001440 PMC11240387

[15] Wolff Sagy Y Ramot N Battat E Arbel R Reges O Dicker D . Glucagon-like peptide-1 receptor agonists compared with bariatric metabolic surgery and the risk of obesity-related cancer: an observational, retrospective cohort study. EClinicalMedicine. (2025) 83:103213. doi: 10.1016/j.eclinm.2025.103213 40599584 PMC12208935

[16] Ailawadi S Murphy JE Storandt MH Mahipal A . Glucagon-like peptide-1 receptor agonist use and pancreatic cancer risk in patients with chronic pancreatitis. Cancers. (2026) 18:179. doi: 10.3390/cancers18020179 41595103 PMC12839127

[17] Alchirazi KA El Telbany A Baliss M Alsabbagh M Kiwan W Bilal M . S5 GLP-1 receptor agonists and pancreatic cancer risk in drug-naive patients with type 2 diabetes. Am J Gastroenterol. (2024) 119:S3–4. doi: 10.14309/01.AJG.0001028388.64456.1E 39449940

[18] Brito JP Herrin J Swarna KS Singh Ospina NM Montori VM Toro-Tobon D . GLP-1RA use and thyroid cancer risk. JAMA Otolaryngol Head Neck Surg. (2025) 151:243–52. doi: 10.1001/JAMAOTO.2024.4852 39847346 PMC11907303

[19] Wang L Berger NA Kaelber DC Xu R . Association of GLP-1 receptor agonists and hepatocellular carcinoma incidence and hepatic decompensation in patients with type 2 diabetes. Gastroenterology. (2024) 167:689–703. doi: 10.1053/j.gastro.2024.04.029 38692395 PMC12294230

[20] Radwan RM Lu Y Dai H George TJ Guo Y Guo J . GLP-1 RA use and survival among older adults with cancer and type 2 diabetes. JAMA Netw Open. (2025) 8:e2521887. doi: 10.1001/JAMANETWORKOPEN.2025.21887 40679827 PMC12274974

[21] Harris M Harvie M Renehan AG . Glucagon-like peptide-1 (GLP-1) receptor agonists and cancer prevention: methodological pitfalls in observational studies. Cancers. (2025) 17. doi: 10.3390/cancers17091451 40361378 PMC12070977

[22] Hicks BM Yin H Yu OHY Pollak MN Platt RW Azoulay L . Glucagon-like peptide-1 analogues and risk of breast cancer in women with type 2 diabetes: population based cohort study using the UK Clinical Practice Research Datalink. BMJ (Online). (2016) 355. doi: 10.1136/BMJ.I5340 27797785

[23] Morales DR Bu F Viernes B Duvall SL Matheny ME Simon KR . Risk of thyroid tumors with GLP-1 receptor agonists: a retrospective cohort study. Diabetes Care. (2025) 48:1386. doi: 10.2337/DC25-0154 40465422 PMC12281980

[24] Henney AE Heague M Riley DR Hydes TJ Anson M Alam U . Synergistic associations of metformin and GLP-1 receptor agonist use with adiposity-related cancer incidence in people living with type 2 diabetes. Diabetes Obes Metab. (2025). doi: 10.1111/DOM.70267 41178701 PMC12803636

[25] Ayoub M Aibani R Dodd T Ceesay M Bhinder M Faris C . Risk of esophageal and gastric cancer in patients with type 2 diabetes receiving glucagon-like peptide-1 receptor agonists (GLP-1 RAs): a national analysis. Cancers (Bsl). (2024) 16:3224. doi: 10.3390/cancers16183224 PMC11430483 39335195

[26] Dankner R Murad H Agay N Olmer L Freedman LS . Glucagon-like peptide-1 receptor agonists and pancreatic cancer risk in patients with type 2 diabetes. JAMA Netw Open. (2024) 7:E2350408. doi: 10.1001/jamanetworkopen.2023.50408 38175642 PMC10767614

[27] Wang CH Chang WC Chen HY Chen PH Lin MH Lee CH . Association of dual SGLT-2 inhibitor and GLP-1 receptor agonist therapy with colon cancer risk in post-polypectomy patients with diabetes: a target trial emulation. Diabetol Metab Syndrome. (2026) 18:109–. doi: 10.1186/S13098-026-02151-X 41906175 PMC13154574

[28] Bezin J Gouverneur A Penichon M Mathieu C Garrel R Hillaire-Buys D . GLP-1 receptor agonists and the risk of thyroid cancer. Diabetes Care. (2023) 46:384–90. doi: 10.2337/DC22-1148 36356111

[29] Kim M Kim SC Kim J Kim BH . Use of glucagon-like peptide-1 receptor agonists does not increase the risk of cancer in patients with type 2 diabetes mellitus. Diabetes Metab J. (2024) 49:49–59. doi: 10.4093/DMJ.2024.0105 39443282 PMC11788542

[30] Levy S Attia A Elshazli RM Abdelmaksoud A Tatum D Aiash H . Differential effects of GLP-1 receptor agonists on cancer risk in obesity: a nationwide analysis of 1.1 million patients. Cancers (Bsl). (2025) 17:78. doi: 10.3390/cancers17010078 PMC11720624 39796706

[31] Dai H Li Y Lee YA Lu Y George TJ Donahoo WT . GLP-1 receptor agonists and cancer risk in adults with obesity. JAMA Oncol. (2025) 11. doi: 10.1001/JAMAONCOL.2025.2681 40839273 PMC12371547

[32] Wang C Wu Z Zhou J Cheng B Huang Y . Semaglutide, a glucagon-like peptide-1 receptor agonist, inhibits oral squamous cell carcinoma growth through P38 MAPK signaling pathway. J Cancer Res Clin Oncol. (2025) 151:103. doi: 10.1007/S00432-025-06154-5 40055197 PMC11889073

[33] Pollack R Stokar J . Long-term glucagon-like peptide 1 receptor agonist use is not associated with increased risk of thyroid cancer in adults with type 2 diabetes. Diabetes Metab Res Rev. (2025) 41. doi: 10.1002/DMRR.70104 41182904 PMC12582397

[34] Farooq Wali A Rangraze I Khan S Mufti UB El-Tanani M Rizzo M . Reassessing cancer risk with GLP-1 receptor agonists: a comprehensive meta-analysis of gastrointestinal Malignancies. Front Pharmacol. (2026) 17:1736380. doi: 10.3389/fphar.2026.1736380 41743116 PMC12929497

[35] Ateiwi YA Mahmood R Wong HJ Low CE Yau CE Lee AB . Glucagon-like peptide-1 receptor agonists and the risk of obesity-related cancers: a systematic review and meta-analysis. Diabetes Res Clin Pract. (2026) 234. doi: 10.1016/j.diabres.2026.113158 41722869

[36] Pasternak B Wintzell V Hviid A Eliasson B Gudbjörnsdottir S Jonasson C . Glucagon-like peptide 1 receptor agonist use and risk of thyroid cancer: Scandinavian cohort study. BMJ. (2024) 385. doi: 10.1136/BMJ-2023-078225 38683947 PMC11004669

[37] Zhong Y Wu T Khan NU . Association between GLP-1 receptor agonists as a class and colorectal cancer risk: a meta-analysis of retrospective cohort studies. BMC Gastroenterol. (2025) 25:614. doi: 10.1186/s12876-025-04211-4 40847331 PMC12372225

[38] Dalbeni A Vicardi M Natola LA Cattazzo F Auriemma A Lombardi R . Glucagon-like peptide-1 receptor agonists and hepatocellular carcinoma prevention: a meta-analysis and clinical decision framework. Cancer Med. (2025) 14:e71434. doi: 10.1002/CAM4.71434 41388641 PMC12701129

[39] Simancas-Racines D Campuzano-Donoso M Román-Galeano NM Zambrano-Villacres R Memoli P Verde L . Obesity and endometrial cancer: biological mechanisms, nutritional strategies, and clinical perspectives. Food Agric Immunol. (2025) 36. doi: 10.1080/09540105.2025.2510961 37339054

[40] Yen TT Hsieh TYJ Lee GY Toy EP Wei JCC Tanner EJ . GLP-1 receptor agonists plus progestins and endometrial cancer risk in nonmalignant uterine diseases. JAMA Netw Open. (2026) 9:e2558205–e2558205. doi: 10.1001/JAMANETWORKOPEN.2025.58205 41665904 PMC12892152

