# Supplementary material for: Cancer outcomes and biological mechanisms among patients with type 2 diabetes mellitus using glucagon-like peptide-1 receptor agonists: a systematic review and meta-analysis
Source: Front Oncol. 2026 Jul 14;16:1859759. doi: 10.3389/fonc.2026.1859759 (PMC13407199; doi:10.3389/fonc.2026.1859759)
Supplement: Supplementary file 2 [file Table2.docx]

## 2.4 Search Strategy (Sample for PubMed)

(("GLP-1 Receptor Agonist" OR "GLP-1RAs" OR liraglutide OR semaglutide OR dulaglutide OR exenatide OR lixisenatide OR tirzepatide)) AND (cancer OR malignancy OR neoplasm OR carcinoma OR tumor) AND (Type 2 diabetes OR T2DM)

Search terms adapted for other databases.

WOS

(GLP-1 Receptor Agonist OR GLP-1RAs OR liraglutide OR semaglutide OR dulaglutide OR exenatide OR lixisenatide OR tirzepatide) AND (cancer OR malignan* OR neoplasm* OR tumor*) AND (type 2 diabetes OR T2DM)

Scopus:

TITLE-ABS-KEY ("GLP-1 receptor agonist" OR "GLP-1RA" OR liraglutide OR semaglutide OR dulaglutide OR exenatide OR lixisenatide OR tirzepatide) AND TITLE-ABS-KEY (cancer OR malignan* OR neoplasm* OR carcinoma*) AND TITLE-ABS-KEY ("type 2 diabetes" OR T2DM)

Search period (years covered): 2015 to 31^st^ May, 2026
